# Injections through skin colonized with *Staphylococcus aureus* biofilm introduce contamination despite standard antimicrobial preparation procedures

**DOI:** 10.1038/srep45070

**Published:** 2017-03-23

**Authors:** Yi Wang, Valery Leng, Viraj Patel, K. Scott Phillips

**Affiliations:** 1 United States Food and Drug Administration, Office of Medical Products and Tobacco, Center for Devices and Radiological Health, Office of Science and Engineering Laboratories, Division of Biology, Chemistry and Materials Science, 10903 New Hampshire Ave, Silver Spring, MD, 20993, USA

## Abstract

While surgical site preparation has been extensively studied, there is little information about resistance of skin microbiota in the biofilm form to antimicrobial decontamination, and there are no quantitative models to study how biofilm might be transferred into sterile tissue/implant materials during injections for joint spine and tendon, aspiration biopsies and dermal fillers (DF). In this work, we develop two *in vitro* models to simulate the process of skin preparation and DF injection using pig skin and SimSkin (silicone) materials, respectively. Using the pig skin model, we tested three of the most common skin preparation wipes (alcohol, chlorhexidine and povidone iodine) and found that during wiping they reduced the biofilm bacterial burden of *S. aureus* (CFU cm^−2^) by three logs with no statistically significant differences between wipes. Using the SimSkin model, we found that transfer of viable bacteria increased with needle diameter for 30G, 25G and 18G needles. Transfer incidence decreased as injection depth was increased from 1 mm to 3 mm. Serial puncture and linear threading injection styles had similar transfer incidence, whereas fanning significantly increased transfer incidence. The results show that contamination of DF during injection is a risk that can be reduced by modifying skin prep and injection practices.

The skin is an immune barrier^1^ and ecosystem colonized by diverse microorganisms. Although most of this microbiota is harmless or even beneficial to the host, the presence of pathogenic bacteria can lead to infection if the ecosystem is disturbed^2^. There are a number of skin penetrating medical procedures where antimicrobial skin preparation is essential, such as surgery, injections into joints, spine and tendon (steroids or hyaluronic acid), needle aspiration for biopsies, and injection of dermal fillers (DF) and urethral bulking agents. While surgical site preparation is extensively studied^3^,^4^, less is known about how injection site preparation might reduce the chance of contamination by skin microbiota.

DF are used with increasing frequency in the US (market size of ~$5 billion in 2014^5^). Injections of foreign materials such as DF present additional risk because the initial number of pathogenic bacteria required to overwhelm host defense mechanisms is low^6^. Bacteria can rapidly colonize injectable DF hydrogels and acquire antimicrobial resistance *in vitro*^7^,^8^. When DF biofilm matures, even the highest tolerable doses of antibiotics are often insufficient to eradicate it^9^. Explant is frequently needed, putting the patient at risk for tissue scarring, deformity, and nerve and structural damage. While clinically detectable infections associated with DF injection are normally rare, patients with a compromised immune system (HIV, cancer, immunotherapy, diabetes) have been reported to have infection rates as high as 19%^10^,^11^,^12^. Some minimally biodegradable filler materials have also been reported to have higher rates of delayed-onset infection^13^.

The pathogenesis of implant infection in general is thought to depend on three steps: transfer of bacteria from skin surface to tissue cells beneath, invasion of tissue with evasion of host defenses, and production of toxins^14^. In individuals with co-morbidities, infection may spread without medical intervention^12^,^15^. Clinical detection of biofilm colonized implants is challenging^16^,^17^. The presence of foreign material in the dermis and sub-dermis can lead to sterile abscesses, granulomas, cellulitis or nodules. Infection may have similar signs which appear months or years after injection, varying from erythema, edema, inflammatory nodules and pain/itching to systemic responses^11^. Biopsies of suspect sites are often culture negative, contributing to the belief that most adverse events are due to allergic or foreign body reactions. Positive biopsies have been explained as instances of contamination during sampling^18^. It has also been argued that DF do not favor bacterial growth^19^ because many hydrogels used for DF materials have antifouling properties^20^,^21^,^22^. However, there is growing evidence linking chronic inflammation, device failure and other adverse events with bacterial colonization of tissues and implant materials^23^. The use of molecular diagnostics (PCR, PNA FISH) and advanced imaging (CLSM, SEM) techniques^24^,^25^,^26^ has conclusively demonstrated correlation and causality. Our group has also shown that bacteria can colonize and form biofilm on ultrasoft hydrogels throughout the range of elastic moduli used for most DF^7^. Injection of some materials can create discontinuities that serve as a protected, microenvironmental niche for bacteria.

Since DF are provided sterile, contamination likely occurs when microbiota on the skin are transferred during needle penetration. Hematogenous bacterial spread is also believed to be a potential risk for all implants in general, but likely represents a smaller fraction of infections when precautions are taken^15^,^27^. Even though DF injections are normally considered a “low-risk” nonsurgical intervention, it is suggested that “extreme care” should be taken to prevent initial contamination associated with injection^25^. The fact that injection near infected skin regions is contraindicated because it increases complications also shows the importance of the skin as a source of contamination^28^. There is increasing comprehension that biofilm is the default lifestyle of bacteria^29^,^30^, and recent studies suggest that many bacteria colonizing the skin produce biofilm^31^, which is resistant to dessication^32^. During injection, bacteria in biofilm can be introduced from the dermal matrix. Sebaceous glands have significant biofilm burden^33^. Wounds or dermatitis may also harbor increased bioburden. Another source of contamination is injection through colonized oral or nasal mucosa, which is more likely to have mature biofilm^34^.

Since contamination during injection is the first step in the pathogenesis process, preventative measures may play an important role in reducing infections. Studies have suggested that 20% of resident skin flora are not removed by preoperative skin prep procedures for open incision surgeries^35^, and DF skin prep is less stringent than most surgical site prep procedures. If transfer of bioburden can be reduced, many of the challenges associated with treating colonized implants can be avoided, thereby improving patient experience and increasing antimicrobial stewardship. To reduce contamination, we need to understand 1) how to better prepare the skin to reduce biofilm burden, and 2) how to best perform injection to reduce the transfer of remaining burden. Most current antimicrobial test methods^36^ are based on planktonic efficacy and may be less predictive of performance against bacteria in biofilm. Models have been developed to test skin preparation^37^,^38^,^39^, but do not use biofilm. Bacteria in biofilm are resistant to antimicrobials through a number of mechanisms^40^. It is important to test common preparation methods to determine how well they reduce biofilm burden on an organic matrix like skin. Previous studies have shown reduced antimicrobial efficacy in murine^41^ and pig^42^ skin models. In addition, no studies have been performed to understand how factors such as needle size/shape and injection style affect the potential for contamination. It is important to develop appropriate biofilm models to determine how these factors might affect transfer of bacteria.

The goal of this work was to develop methods for assessing contamination during skin preparation and injection, and to use them to assess practices used in the clinic. To measure the removal of biofilm burden after skin preparation, we developed an *ex vivo* porcine skin explant model. Three commercially available disinfectant preparation pads were tested. To assess how injection techniques affected bacterial transfer, we developed an *in vitro* injection model with artificial silicone skin (SimSkin) of specific thicknesses to represent depth of placement. Injection was simulated using several sizes of needles with common techniques (puncture, threading and fanning). Bacterial transfer for planktonic and biofilm bacteria on skin surfaces was compared.

## Materials and Methods

### Bacterial culture

Green fluorescent protein (GFP) producing *Staphylococcus aureus (S. aureus*) AH2547^43^ provided by Dr. Alexander Horswill (Department of Biology, The University of Iowa, Iowa City, IA) was streaked for isolation on a source agar plate (tryptic soy agar (TSA) with 5% Sheep Blood; Remel^TM^, Thermo Scientific^TM^) from frozen stock cultures and incubated overnight (16–18 h) at 37 °C. A dispersed single/double bacterial cell solution was prepared using previously described methods^44^. The concentration was determined to be 10^9^ CFU mL^−1^ by plating on agar plates. The solution was re-suspended to concentrations used in the study. Different inoculum densities (10^2^–10^8^ CFU mL^−1^) were cultured at 37 °C in tryptic soy broth (TSB, Becton Dickinson) overnight to generate biofilm bioburden. For comparison, bioburden from planktonic contamination was introduced to skin surfaces with the same inoculum density for 5 min and followed with a phosphate buffered saline (PBS, pH 7.4, Thermo Scientific^TM^) rinse. Chloramphenicol (10 μg/mL) was added to all culture media to maintain the stability of plasmid^45^ and also inhibit native microorganisms on pig skin. All experiments were replicated in triplicate with three independent *S. aureus* cultures.

### *Ex vivo* porcine skin explant biofilm model for skin preparation

**Figure 1:**
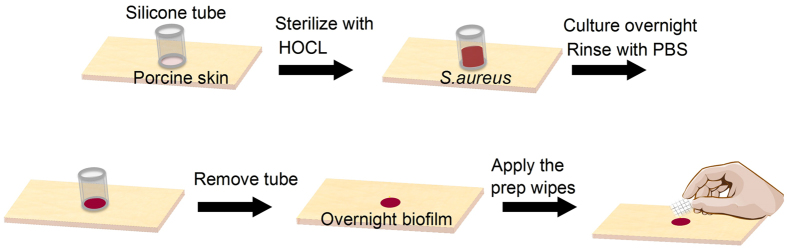
Biofilm model to assess skin preparation.

An *ex vivo* porcine skin explant biofilm model was developed to assess the bioburden removal efficacy of commercially available disinfectant preparation pads and topical treatments (Fig. 1). Porcine skin explants (Pel-freeze Biologicals, Rogers, AR) were grafted and cut to blocks with dimension of ~1in × 1in × 0.5in. Silicone tubing (autoclaved, 6 mm ID, Thomas Scientific, NJ) was cut to 10 mm long and glued to skin blocks (one tube/block). Neutral electrolyzed water (NEW) (Aquaox, Fontana, CA) was used to sterilize skin pieces^46^. Diluted NEW solutions (HOCl, 80 mg/l) were applied twice (15 min/each) to the skin glued with tubing. The whole blocks were rinsed with sterile PBS and stored sterile.

Overnight biofilms were generated in each tube by inoculating with 200 μL 10^5^ CFU mL^−1^ bacteria. Inoculated tubes were rinsed 3x with PBS and removed from the skin. Skin was wiped with preparation pads and rinsed with PBS after 10 s. Three pad types (alcohol, chlorhexidine and povidone-iodine) were applied to biofilms on porcine skins with controlled force and direction (4 lateral wipes with return stroke). A coverslip was placed on the wiped location and the assembly attached to a glass slide. Confocal Laser Scanning Microscopy (CLSM, Leica SP8, Leica Microsystems, Germany) was used to acquire images of bioburden before and after wiping. CLSM images were collected with 485 nm excitation/535 nm emission. Simulated fluorescence projections through the biofilm were generated using the Leica LAS software, from which live bacteria were enumerated for nine samples (9 locations/sample). All experiments were replicated in triplicate with three independent *S. aureus* cultures.

### SimSkin *in vitro* injection model

**Figure 2:**
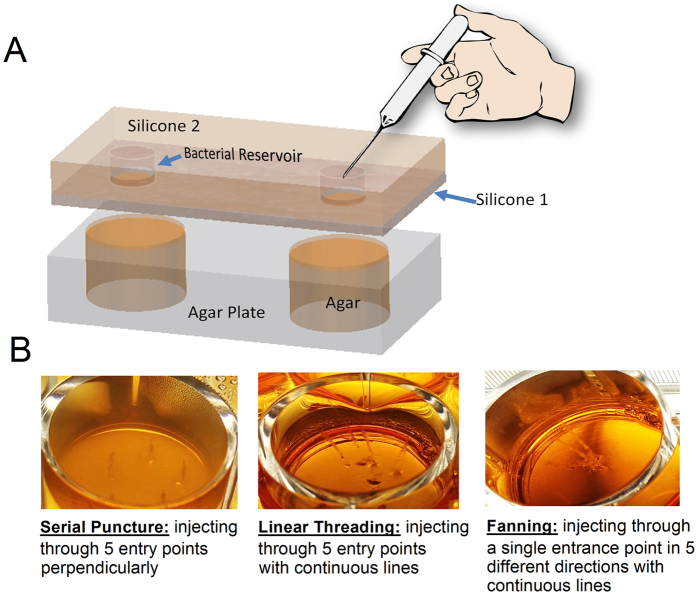
Simulated injection model (**A**) and trails in agar showing different injection techniques (**B**).

The filler injection-mimicking artificial skin system (Fig. 2A) was prepared by laminating an epidermis and dermis-simulating layer (Silicone 1, SIMSKIN, Chicago, IL, - 1, 1.8 or 3 mm thickness silicone elastomer containing reinforcing fibers) to an overlayer of 8 mm thick Silicone 2. The epidermis layer (0.1 mm) is made of a high tear strength elastomer. Silicone 2 was punched with 6 mm wide holes as a reservoir for bacteria and the assembly was cut to fit a 12-well plate. Silicone 2 created the reservoir for bacterial inoculation, while the Slicone 1 underlayer served as the skin mimetic material in testing.. Modified TSB agar (with 5:1 gelatin:TSB by mass) was filled in a 12-well plate. The model skin system was held to the agar plate underneath by directly attaching Silicone 1 to the modified TSB agar in order to assess different injection techniques (needle size and shape, serial puncture, linear threading, and fanning) (Fig. 2B). A sterile liquid with similar elastic properties to DF (DF simulant) was used for all injections. Bacterial reservoirs were inoculated with 200 μL bacteria (10^2^, 10^5^, and 10^8^ CFU mL^−1^) to mimic clinical (CLIN), normal (NORM) and contaminated (CONT) bioburden levels for injections^47^,^48^,^49^. DF simulant was also injected for each condition to serve as a control. Silicone 1 with thickness of 1, 1.8 and 3 mm was used to model injection in the superficial dermis, deep dermis and subcutaneous, respectively. To compare different injection conditions, 18, 25, and 30 gauge sharp needles (1.5in., Becton Dickinson) and 25 gauge blunt-tipped microcannula (1.5in., DermaSculpt) were tested. Three parallel conditions were applied and repeated with 9 independent *S. aureus* cultures. To mimic the dermal filler injection, 5 entries were performed through each bacterial reservoir and model skin to the agar beneath (Fig. 2B). After injection, the model skin system was removed, and the agar plates were incubated at 37 °C for 24–48 h. To validate the artificial skin model, porcine skin was grafted to ~2 mm thick and cut and glued to sterile Silicone 2. The same sterilization and injection protocols were followed as for the silicone model. Bacterial transfer was evaluated after overnight culture. Transfer incidence and error bars were generated by averaging the possibilities of transfer from each agar plate for the nine sets of injection experiments.

### Scanning electron microscopy

Disinfectant preparation pads were air dried overnight and sputter coated with gold/palladium (10 nm, EMS150T, EMS, PA), and imaged using SEM (JSM-6390 LV, JEOL, MA) at a voltage of 5 kv.

### Statistical analysis

Statistical analysis was performed using one-way analysis of variance (ANOVA) to detect the presence of statistically significant differences (P < 0.05) between groups. All experiments were repeated at least three times.

## Results

### Efficacy of skin preparation wipes for reduction of biofilm bacterial burden

**Figure 3:**
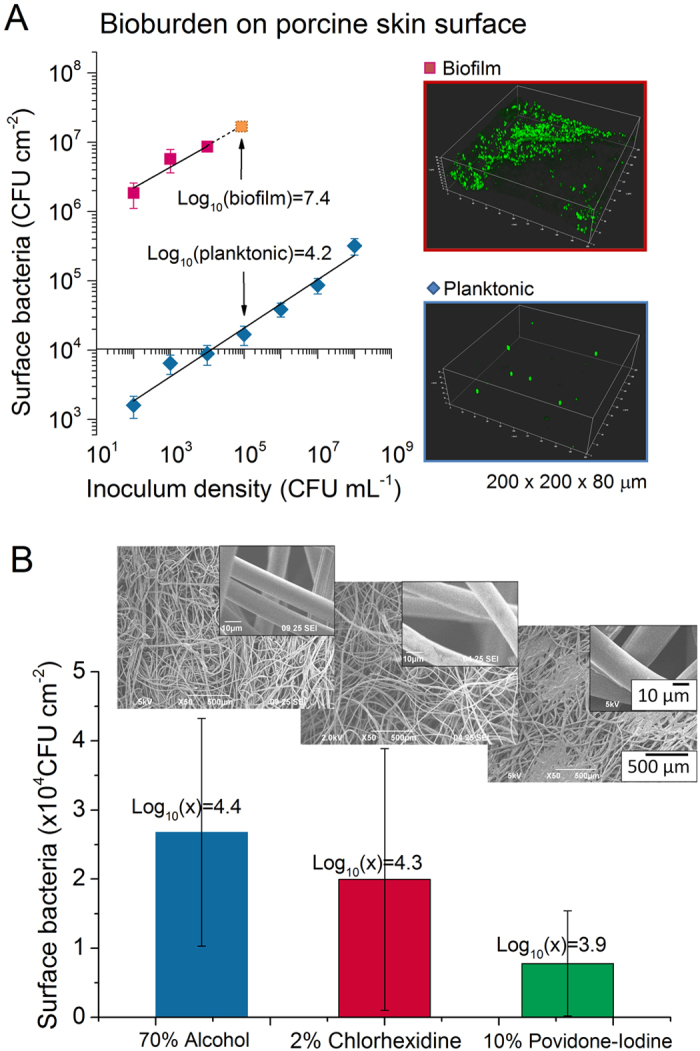
Porcine skin planktonic and biofilm bioburden (CFUcm^−2^) before (**A**) and after (**B**) skin preparation. CLSM images (Insert, A) of the biomass formed with inoculum density of 10^5^ CFU mL^−1^. SEM images (Insert, B) show similar textures of preparation pads.

Porcine skin surface was inoculated with *S. aureus* (10^5^ CFU mL^−1^) and cultured overnight for biofilm formation. Initial bioburden was quantified with image analysis. Before applying the preparation wipes (Fig. 3A), no significant difference was found in the amount of bioburden formed with the same inoculum. Using a log-log transformation to compare bioburden in planktonic and biofilm forms, the increase of surface bioburden was linear (slope = 0.3487, R^2^ = 0.9975), suggesting that the relationship between inoculum densities and both planktonic and biofilm burden fit closely to power law. For planktonic inoculation, the CLIN, normal NORM and contaminated CONT bioburden levels result in surface bacterial densities of (0.16 ± 0.056) × 10^4^, (1.7 ± 0.53) × 10^4^, and (31 ± 8.5) × 10^4^ cm^−2^. For biofilm, the bioburden left on the skin surface also increased with inoculum densities (10^2^, 10^3^, 10^4^ CFU mL^−1^) corresponding to surface densities of (185 ± 74.1) × 10^4^, (576 ± 212) × 10^4^ and (866 ± 106) × 10^4^ cm^−2^. An assumption of linearity was used for the 10^5^ CFU mL^−1^ inoculum. In general, there was a 3 log difference in cell counts between planktonic burden and overnight biofilm burden.

After skin preparation with disinfectant wipes, greater spatial heterogeneity of live bacteria was observed on skin surfaces. Less bacteria remained on the 10% povidone-iodine treated skin (log_10_ (B) = 3.9, Fig. 3B). No significant difference was seen between 70% alcohol (log_10_ (B) = 4.4) and 2% chlorhexidine (log_10_ (B) = 4.3) treated skin. Overall, there was less than one log difference between the three disinfectant wipes. SEM images (Fig. 3B) showed that all wipe textures and fiber widths were similar.

### Transfer of bacterial biofilm burden during injection

**Figure 4:**
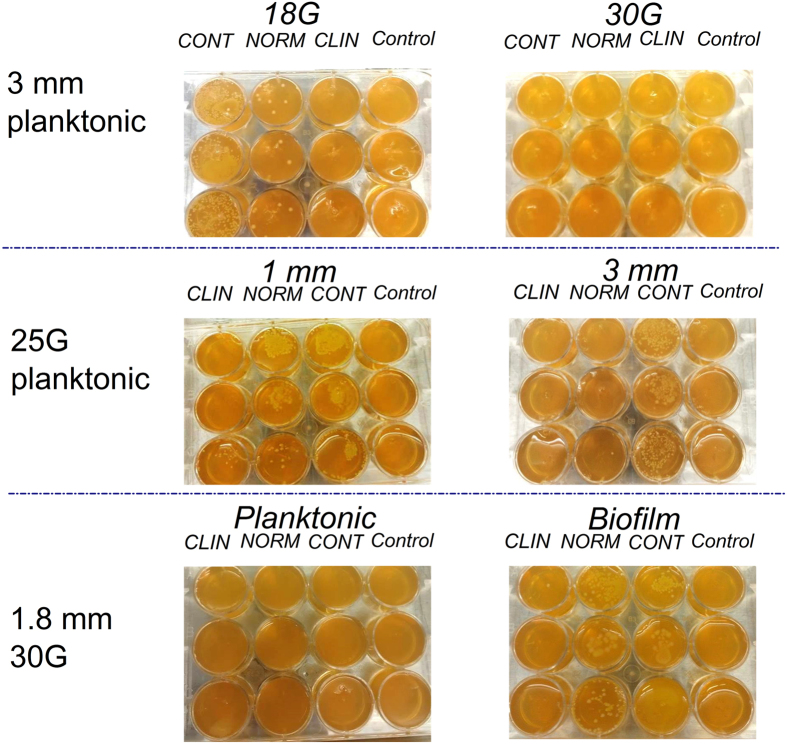
Representative images of bacterial transfer for different injection conditions with the SimSkin model (needle size, depth of placement and planktonic/biofilm bacterial contamination).

**Figure 5:**
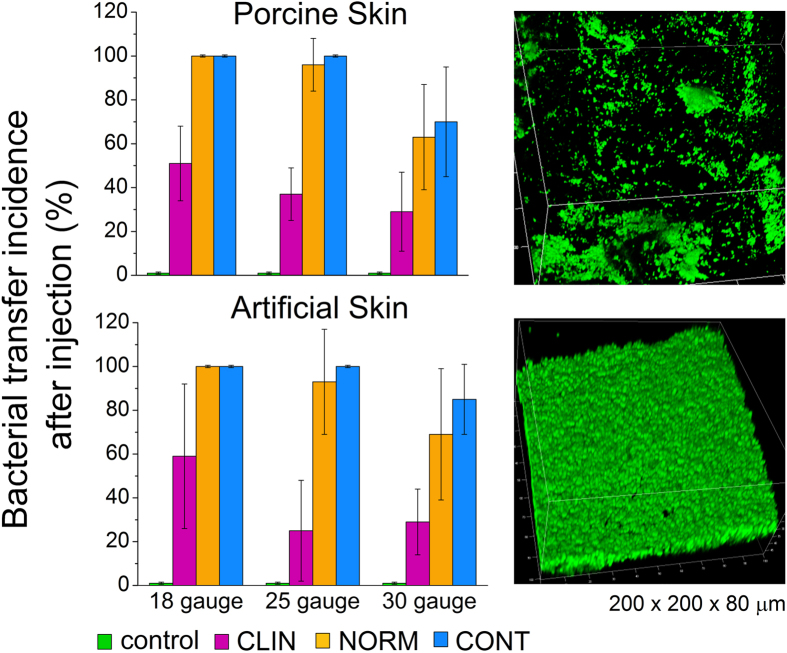
Bacterial transfer incidence of porcine skin (2 mm) and SimSkin (1.8 mm) with 18G, 25G and 30G needles and biofilm as initial bioburden. Confocal fluorescence image stacks (200 μm × 200 μm × 80 μm) of biofilm formed from inoculum of 10^5^ CFU mL^−1^.

**Figure 6:**
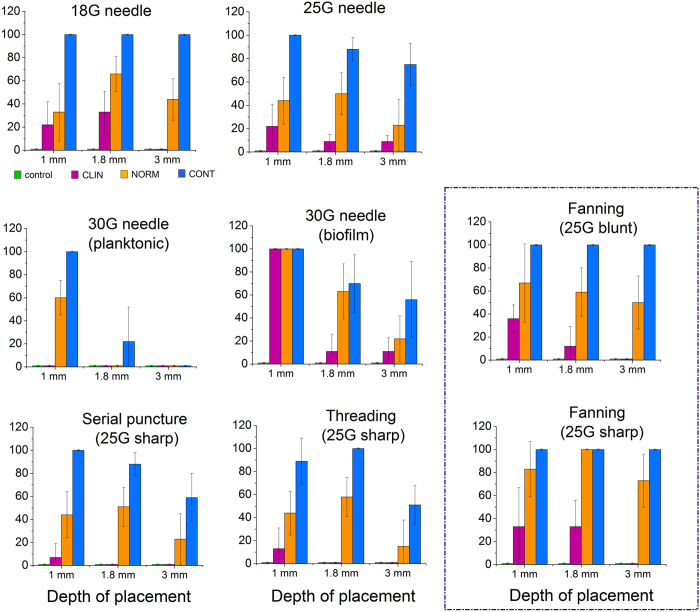
Quantification of bacterial transfer under different injection conditions (needle size/shape, depth of placement, injection techniques and initial bioburden).

Representative images (Fig. 4) show how bacterial colonies that grew at the site of contamination were used to track bacterial transfer during injection. Porcine skin was initially used but was later replaced by SimSkin which compared favorably for transfer incidence for a limited subset (Fig. 5). The SimSkin format was used to assess how the transfer incidence depends on: needle size – G (gauge, larger gauge = smaller needle size), injection depth, growth forms of bacteria (planktonic vs. biofilm) and common DF injection techniques (serial puncture, threading and fanning) (Fig. 6). All of the experiments were done with serial puncture except for those that compared other injection techniques.

The results for all conditions are summarized in the graphs in Fig. 6. For all experiments, transfer incidence increased with needle size and starting bioburden and decreased with injection depth. Only 30G needles were tested with both planktonic and biofilm bioburdens. Biofilm transfer incidence was much higher than planktonic transfer incidence. The transfer incidence for serial puncture and threading was similar at all depths and bioburden, while fanning had significantly higher transfer rates for all depths and bioburdens. There was no significant increase in transfer incidence for fanning with a blunt-tipped microcannula vs. sharp tip needle.

## Discussion

### Skin preparation

The current clinical practice for DF injection involves preparing the site of injection with an antiseptic wipe^50^. Three of the most common antiseptics, isopropyl alcohol (IPA), chlorhexidine (CHG) and povidone iodine (PI), are lethal to bacteria through different mechanisms^51^. Some reports studying the performance of these disinfectants against biofilm have shown decreased effectiveness^52^,^53^,^54^,^55^. In this work, all three antiseptics achieved a similar ~3 log reduction of biofilm burden after wiping. While the porcine skin explant biofilm model did show statistically significant differences among bioburden reduction by various antiseptic wipes (P < 0.05), the ≤½ log difference is not practically different with regards to effectiveness^42^.

The porcine skin format shows that the medium of skin, due to the surface roughness, absorption of liquid, and abundance of organic material, may play a role in neutralizing antiseptics to some extent and possibly further protecting biofilm. Confocal microscopy images of biofilm on the skin (Fig. 5) showed that it was heterogeneous because of surface irregularities. Biofilm bacteria in those cracks and crevices may be more resistant to the mechanical force of wiping. This may explain in part why the reduction in bioburden was only 3 logs for all preparations. Depending on the location^56^, flora on skin can exceed 1.6 × 10^6^ aerobic CFU and >0.9 × 10^6^ fungal colonies. Considering these levels, a 3 log reduction still leaves significant bioburden.

### Injection

The SimSkin testing showed that transfer rarely occurred at all with the smallest 30G needle except at the 1 mm depth, while it increased with diameter for the 25G and 18G needles. The needle tip shape (sharp vs. blunt-tipped microcannula) did not make a significant difference in outcome. These results are promising because it is reported that the blunt microcannula technique may produce less brusing^57^ in some situations. Of particular importance, the incidence of biofilm burden transfer at 1 mm depth and high inoculum (CONT) was 100% for all needle sizes. Even for biofilm with the smallest needle size and lowest initial inoculum (CLIN), the incidence of transfer was high–100% at 1 mm, 33% at 1.8 mm, and 10% at 3 mm. Higher transfer incidence for biofilm was likely a result of the increased density and protective EPS matrix. The barrier function of the skin depended on the distance of cutaneous tissue through which the needle traveled.

Moreover, the injection technique that shared the same entry points (fanning) resulted in higher transfer probability than techniques with separate entry points (serial puncture and threading). The transfer incidence was the highest for fanning, with the CONT bioburden level resulting in 100% probability for all three injection depths, and even the NORM bioburden resulting in 70–100% probability. Although fanning is reported as a common technique for filler injection^58^, based on the results obtained here, further evaluation is warranted.

### Putting it all together

While *in vitro* simulation is a limitation of this work, the reduced variability in test conditions achieved through these models provides significant insight as to where further investigation is necessary *in vivo*. Combining the information from skin preparation and injection testing, it becomes clear that the chance of DF contamination is likely in many scenarios despite skin preparation. Considering a best case scenario (planktonic bacteria at the CLIN bioburden level, 10^2^ CFU cm^−2^), for shallow (1 mm) injections, the only way to completely eliminate the probability of bioburden transfer is to use a very small (30G) needle and avoid the fanning style. The same is mostly true for intermediate depth (1.8 mm) injections, with exception of serial puncture.

Given the limitation that in most cases clinicians may need to use a particular needle/injection style to achieve a desired outcome, there are still some ways to ensure safer DF use. The first is improved antiseptic strategies to decrease the biofilm burden. Greater wipe time and multiple wipe steps are simple measures to improve skin preparation. More sophisticated ways to improve performance might include novel antimicrobials to help penetrate or dissolve biofilm. Recent studies have shown that infection is more likely when a bolus of biofilm is injected than individual planktonic cells^34^,^54^,^59^. Filler materials that resist colonization or contain anti-biofilm components may also interfere with colonization.

Methods that are developed to improve skin preparation for DF injection may also benefit other types of injection where infection is a risk. The most frequent complication of joint, tendon and muscle injections is infection^60^. In the specific case where injections are given pre- or post-operatively with arthroplasty, they are also associated with an increased risk of prosthetic joint infection^61^. Prosthetic joint infection is a devastating complication for patients, increasing both morbidity and mortality^62^. In particular, orthopedic oncology with its higher infection incidence stands to benefit^63^.

### Disclaimer

The mention of commercial products, their sources, or their use in connection with material reported herein is not to be construed as either an actual or implied endorsement of such products by the Department of Health and Human Services. The findings and conclusions in this article have not been formally disseminated by the U.S. Food and Drug Administration and should not be construed to represent any Agency determination or policy.

## Additional Information

**How to cite this article**: Wang, Y. *et al*. Injections through skin colonized with *Staphylococcus aureus* biofilm introduce contamination despite standard antimicrobial preparation procedures. *Sci. Rep.* **7**, 45070; doi: 10.1038/srep45070 (2017).

**Publisher's note:** Springer Nature remains neutral with regard to jurisdictional claims in published maps and institutional affiliations.
